# Effectiveness and safety of Glucosamine, chondroitin, the two in combination, or celecoxib in the treatment of osteoarthritis of the knee

**DOI:** 10.1038/srep16827

**Published:** 2015-11-18

**Authors:** Chao Zeng, Jie Wei, Hui Li, Yi-lun Wang, Dong-xing Xie, Tuo Yang, Shu-guang Gao, Yu-sheng Li, Wei Luo, Guang-hua Lei

**Affiliations:** 1 Department of Orthopaedics, Xiangya Hospital, Central South University, Changsha, Hunan Province, China, 410008; 2 Health Management Center, Xiangya Hospital, Central South University, Changsha, Hunan Province, China, 410008; 3 Department of Epidemiology and Health Statistics, School of Public Health, Central South University, Changsha, Hunan Province, China, 410008

## Abstract

This study aimed to investigate the effectiveness and safety of glucosamine, chondroitin, the two in combination, or celecoxib in the treatment of knee osteoarthritis (OA). PubMed, Embase and Cochrane Library were searched through from inception to February 2015. A total of 54 studies covering 16427 patients were included. Glucosamine plus chondroitin, glucosamine alone, and celecoxib were all more effective than placebo in pain relief and function improvement. Specifically, celecoxib is most likely to be the best treatment option, followed by the combination group. All treatment options showed clinically significant improvement from baseline pain, but only glucosamine plus chondroitin showed clinically significant improvement from baseline function. In terms of the structure-modifying effect, both glucosamine alone and chondroitin alone achieved a statistically significant reduction in joint space narrowing. Although no significant difference was observed among the five options with respect to the three major adverse effects (withdrawal due to adverse events, serious adverse events and the number of patients with adverse events), the additional classical meta-analysis showed that celecoxib exhibited a higher rate of gastrointestinal adverse effect comparing with the placebo group. The present study provided evidence for the symptomatic efficacy of glucosamine plus chondroitin in the treatment of knee OA.

In recent years, there has been a fierce controversy over the efficacy of glucosamine, chondroitin, or the two in combination in the treatment of knee osteoarthritis (OA)^1^. The recommendations given by the American College of Rheumatology conditionally do not advocate the use of glucosamine and chondroitin in the treatment of knee OA^2^. The clinical practice guideline of American Academy of Orthopaedic Surgeons also provides strong evidence that the use of glucosamine and chondroitin cannot be recommended^3^. Similarly, the 2014 Osteoarthritis Research Society International (OARSI) guidelines conditionally do not recommend the use of them^3^. However, despite these available guidelines, glucosamine and chondroitin, sold as over-the-counter products in many cases, are among the most popular dietary supplements in America^5^,^6^. Thus, if glucosamine and chondroitin are indeed useless, a great deal of money is being wasted. Meanwhile, in another respect, American Food and Drug Administration (FDA) had approved celecoxib as the first specific inhibitor of cyclo-osygenase-2 (COX-2) in December of 1998. Therefore, celecoxib became the most commonly prescribed inhibitor against COX-2. Out of concerns on adverse effects (AEs), other kinds of specific inhibitor of COX-2, such as etoricoxib, refecoxib, etodolac, valdecoxib and lumiracoxib have not yet passed the certification of FDA. Celecoxib is thereby the only option of selective non-steroidal anti-inflammatory drug (NSAID) currently available in America and is recommended or conditionally recommended by all the three guidelines mentioned above^3^,^4^,^5^,^7^,^8^,^9^.

A recent randomized controlled trial (RCT) conducted by Hochberg *et al.*^10^ suggested that glucosamine plus chondroitin showed comparable efficacy over celecoxib in patients with painful knee OA, with a good safety profile. The effect of celecoxib on the treatment of OA in comparison with placebo had already been proved by our previous network meta-analysis^11^, but the efficacy of glucosamine and chondroitin, especially the two in combination, in comparison with placebo, still remains uncertain. A network meta-analysis published in 2010 concluded that glucosamine, chondroitin, and the two in combination did not relieve joint pain or exerted an impact on narrowing of joint space when compared with placebo, and therefore health authorities and health insurers should not cover the costs of these preparations^12^. However, the evidence was limited because only ten trials were included in their study and only one direct comparison (the combination group versus placebo) was available at that time. In addition, celecoxib, another widespread used oral agent in the treatment of OA, was not included in their Bayesian Network Model^12^. As a matter of fact, there is still a fierce controversy on whether glucosamine and chondroitin are effective for OA patients.

The method of Bayesian network meta-analysis integrates all direct and indirect evidences with respect to the relative medical effects; in this regard, it offers a coherent and unified approach to analyze all RCTs^11^,^13^,^14^,^15^,^16^,^17^,^18^. For the present study, the combination of direct and indirect evidences is an effective way to enhance estimate accuracy, as it can narrow the width of confidence interval compared to direct estimate alone^19^. On the basis of existing evidences, this study conducted a network meta-analysis of RCTs to examine the safety and efficacy of glucosamine, chondroitin, the combination of the two, or celecoxib in the control of knee OA.

## Methods

### Literature search

Electronic database including Cochrane Library, PubMed and Embase were searched through based on logic combinations of keywords and in-text words that are associated with OA to extract concerned RCTs and interventions conducted till February 2015 (see Appendix 1). Meanwhile, the following websites were also search through in the manner of periodic scrutiny in order to track unpublished and ongoing studies: the World Health Organization International Clinical Trials Registry (ICTRP) (http://apps.who.int/trialsearch/Default.aspx) and ClinicalTrials.gov (http://www.clinicaltrials.gov/). In addition, reviews and references listed in the retrieved articles were examined manually.

### Study selection

This network meta-analysis is subject to the following inclusion criteria: (1) RCTs; (2) studies about knee OA patients; (3) studies containing at least two of the following treatments: 200 mg/day oral celecoxib, glucosamine, chondroitin, combination of glucosamine and chondroitin, and placebo; (4) studies reporting the pain, function, structure-modifying effect or the side effect outcomes of patients; (5) English literature; (6) availability of data. Secondary studies, such as some combined data analyses of RCTs, were ignored.

### Quality assessment

The Cochrane’s risk of bias table was used to determine the methodological quality of each trial^20^. A total of seven potential risks of bias were evaluated: random sequence generation, allocation concealment, blinding of participants, blinding of outcome assessment, incomplete outcome data, selective reporting and other bias. Studies involving three or more high risks of bias were considered as poor methodological quality.

### Outcome measure

This network meta-analysis aimed to reach the following primary outcome: the effectiveness of pain relief and function improvement from the baseline to the end of treatment by using 200 mg/day oral celecoxib, or glucosamine, or chondroitin, or combination of glucosamine and chondroitin, or placebo for knee OA. If multiple pain scales were reported by any study, the highest one on the hierarchy of pain scale was taken referencing to the suggestion of Jüni *et al.*^21^. The function subscale of Western Ontario and McMaster Universities Arthritis Index (WOMAC) was adopted to assess function improvement. If the WOMAC function was not reported, either Lequesne Index, WOMAC total or other relevant measurement scales would be applied instead. Since different scales were used for the pain and function assessment of included studies, the standard mean difference (SMD) was calculated to determine the differences between various treatment arms for pain relief and function improvement. If the value of SMD is negative, it suggests a better treatment effect in terms of function improvement and pain relief. We back transformed effect sizes (SMD) for pain to differences on a 10 cm visual analogue scale (VAS) based on a median pooled SD of 2.5 cm reported in large scale trials in osteoarthritis that used a similar visual analogue scale for pain. We also back transformed SMD for function to a standardized WOMAC function score ranging from 0 to 10 on the basis of a median pooled SD of 2.1 units^22^. The absolute changes from the baseline of pain and function were also evaluated in the classic meta-analysis. A change of 2 points on the scale of 0 to 10 was considered as clinically significant improvement^23^,^24^. The mean difference (MD) was used to evaluate the joint width narrowing of different treatments. A positive value of MD indicates a smaller joint width narrowing and a better treatment effect.

Safety and tolerability of all included studies were investigated based on the number of patients who withdrew from treatment because of adverse events (AEs) and who experienced serious AEs (SAEs), as well as the number of patients who experienced AEs by network meta-analysis. This study did not define any uniform standard for SAEs, and the definitions given in the original studies were respected. Meanwhile, 6 types of AEs were compared according to pairwise meta-analysis: gastrointestinal (GI) AEs, cardiovascular (CV) AEs, central nervous system (CNS) AEs, musculoskeletal (MU) AEs, infections, and skin AEs. The classification standard of the AEs was described in a former study^11^.

### Statistical analysis

A network meta-analysis Bayesian random effect model was established to examine the overall effect size for different treatment options of knee OA. Network meta-analysis integrates both direct and indirect evidences in all primary trials, which is commonly seen as its main advantage^11^,^25^. The statistical method of this study had been elaborated in detail in our earlier researches^11^,^13^,^14^. In the Bayesian method, the prior probability distribution needs to take into account relevant prior information. Vague prior (mean 0, variance 10000) distributions were used throughout this study to allow data to drive inferences. The Markov Chain Monte Carlo method in the WinBUGS software (version 1.4.3, MRC Biostatistics Unit, Cambridge, UK) was applied to estimate posterior densities for unknown variables, as described in some earlier studies^18^,^26^,^27^. Then, the random effect model instead of the fixed effect model was applied as the most conservative and appropriate analysis to deal with differences among trials. For random effects, homogeneous variance was assumed in this study. Three Markov chains with different initial values (selected arbitrarily for convergence) ran simultaneously, and 50,000 simulations were generated for each set of initial values. To avoid the burn-in period, the first 10,000 simulations were rejected. The WinBUGS codes for random effect models of multi-arm trials can be found at http://www.mtm.uoi.gr/ and http://www.bristol.ac.uk/social-community-medicine/projects/mpes/ (see Appendix 2, the WinBUGS codes for network meta-analysis). The overall effect sizes (ORs or SMDs or MD) were obtained based on the median of the posterior distribution. The lower and upper limit of the traditional 95% credible interval (95%CI) corresponds to the 2.5th and 97.5th percentiles of the posterior distribution respectively. Significant difference was revealed by the 95%CI, which did not consider 1 for OR, or 0 for SMD and MD. Differences between direct and indirect effect estimates with respect to the same comparison were regarded as inconsistency, which was assessed based on the ratio of two odds rations (*RoR*) from direct and indirect evidences in one loop. If the *RoR* value approaches to 1, it indicates that the two estimates are consistent with each other. Loops with the lower 95%CI limit of *RoR* that does not approach to 1 suggest that there exists statistically significant inconsistency^28^. The fit of the model to data was evaluated by calculating the posterior mean residual deviance. If the mean of the residual deviance is similar to the number of data points of the model, this model fits the data adequately^29^. Network meta-analysis is also possible to generate rankings for all concerned treatments on the basis of the level of effectiveness according to posterior probabilities (first best, second best, third best, etc.). The values of probability were reported as the surface under the cumulative ranking (SUCRA). The best treatment has the SUCRA equal to 100%, while the worst treatment has the SUCRA equal to 0%^28^,^30^. Sensitivity analysis was conducted by excluding studies with poor methodological quality, or studies without commercial support, or studies with patients who were not evaluated with moderate or severe pain (VAS ≥ 3 cm for 0–10 cm scale) at baseline for pain and function. This study also conducted classic pairwise meta-analysis, in which heterogeneity was tested by Q statistics (P ≤ 0.05 was regarded as heterogeneous) and I^2^ statistics. I^2^ statistics mean to measure the percentage of the total variation across various studies (I^2^ ≥ 50% was regarded as heterogeneous). Publication bias was assessed by Begg’s test. P ≤ 0.05 indicates the existence of publication bias^31^.

All statistical analyses were established in the WinBUGS software (version 1.4.3, MRC Biostatistics Unit, Cambridge, UK), R version 3.0.2 (The R Foundation for Statistical Computing) and STATA software (version 11.0, StataCorp, College Station, TX).

## Results

### Study selection and characteristics

**Figure 1:**
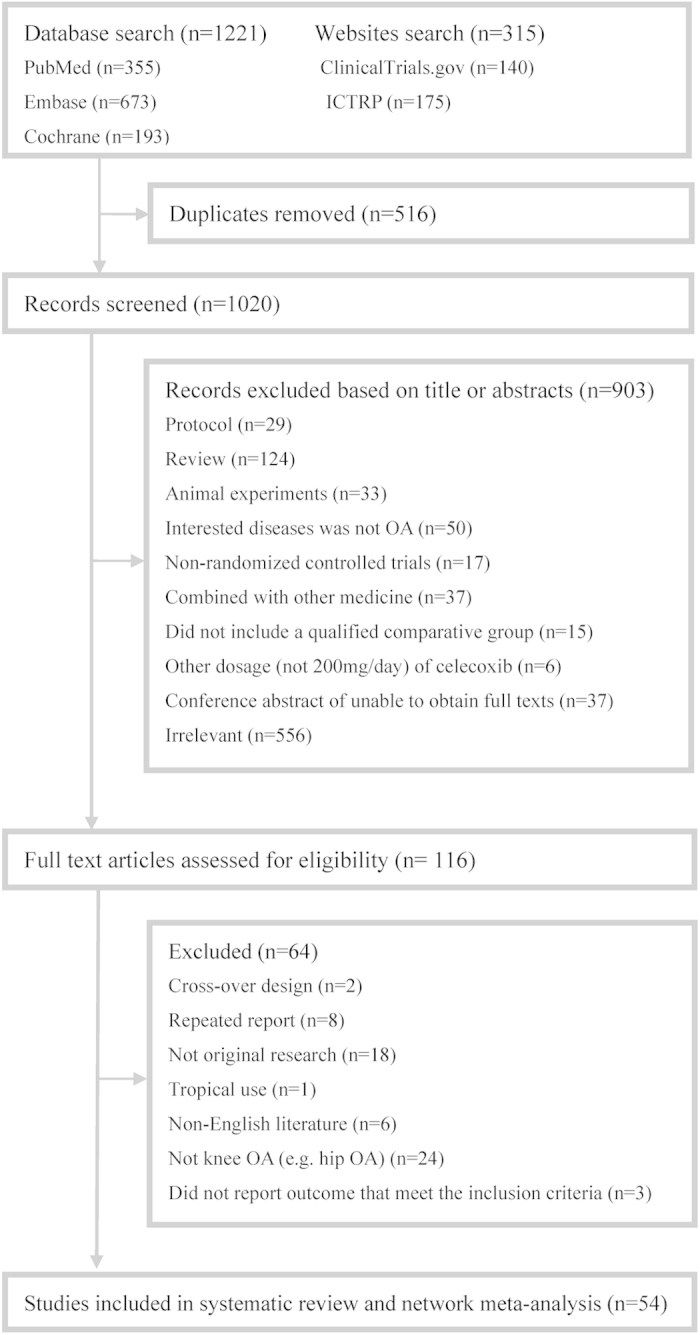
Summary of studies identification and selection.

**Figure 2:**
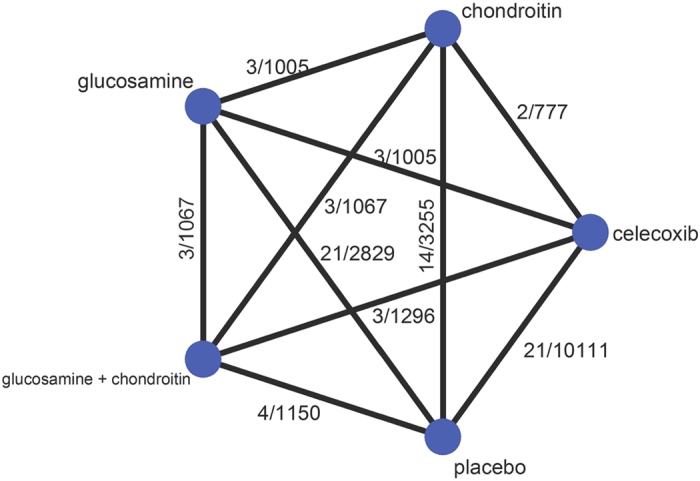
Structure of network formed by interventions and their direct comparisons. The lines between treatment nodes indicate the direct comparisons made within randomized trials. Number showed beside the line represented number of trials/number of participants.

54 studies (Appendix 3) were qualified and included in the present systematic review and network meta-analysis. Figure 1 shows the details of the selection process. It is noteworthy that one study reported two independent trials, and two studies reported different outcomes from one trial. Figure 2 presents the network structure of the comparisons conducted in the present study. The methodological quality for all included trials was evaluated (Appendix 4). A total of 13 studies were considered as poor methodological quality.

### Effects of pain relief

A total of 34 studies reported the change pain score at the last follow-up time point from baseline. Table 1 shows the SMD and the related 95%CI of network meta-analysis in terms of the changed pain score at the last follow-up period. Celecoxib (back transformed difference was −0.68 cm, 95%CI was −0.88 cm to −0.50 cm), glucosamine (−0.50 cm, −0.83 cm to −0.18 cm), chondroitin (−0.45 cm, −0.85 cm to −0.08 cm), and the combination of glucosamine and chondroitin (−0.68 cm, −1.18 cm to −0.15 cm) all showed a significantly better effect on pain relief compared with the placebo group. There was no significant difference in terms of pain relief between any two compared groups. All treatment options met the pre-specified criteria for clinically significant improvement of pain (Appendix 5). Evidence of inconsistency might exist in the comparisons among the loop of celecoxib, glucosamine, chondroitin; and the loop of celecoxib, glucosamine, and the combination of glucosamine and chondroitin. The assessment results of the goodness of fit indicate a general fit with the posterior mean residual deviance equal to 81.76 (71 data points). The probability distribution for each treatment option is presented in Appendix 6. Celecoxib possessed the largest probability to be the best treatment option (81%), followed by the combination of glucosamine and chondroitin (74%), while placebo ranked the last (1%). After excluding studies with poor methodological quality or studies without commercial support, the results did not change significantly. After excluding studies with patients who were not evaluated with moderate or severe pain (VAS ≥ 3 cm on the 0–10 cm scale) at baseline, the results appeared to be similar to the placebo group. Evidence of significant heterogeneity was found in some comparisons, but no publication bias was observed among included studies. (Appendix 7).

### Effects of function improvement

A total of 30 studies reported the change function score at the last follow-up time point from baseline. Table 1 shows the overall SMD and the related 95%CI of network meta-analysis with respect to function improvement. Celecoxib (back transformed difference was −0.59 units, 95%CI was −0.74 units to −0.46 units), glucosamine (−0.40 units, −0.61 units to −0.19 units), and the combination of glucosamine and chondroitin (−0.48 units, −0.80 units to −0.17 units) all showed a significantly better effect on function improvement compared with the placebo group. After back transforming the effect size and the difference between celecoxib and placebo, celecoxib also showed a better effect on function improvement compared with the chondroitin group (back transformed difference was −0.38 units, 95%CI was −0.65 units to −0.06 units). There was no significant difference in terms of function improvement between any other two compared groups. Glucosamine plus chondroitin was the only treatment option meeting the pre-specified criteria for clinically significant improvement of function (Appendix 5). There was no sign of inconsistency between direct and indirect evidences. The assessment of the goodness of fit suggested an adequate fit with the posterior mean residual deviance equal to 64.30 (63 data points). The probability distribution for each treatment is shown in Appendix 6. Celecoxib is most probably to be the best treatment (92%), followed by the combination of glucosamine and chondroitin (72%), while placebo ranked the last (1%). After excluding studies with poor methodological quality or studies without commercial support, the results did not change significantly. Evidence of significant heterogeneity was only found in the comparison between celecoxib and glucosamine, and no publication bias was observed among included studies. (Appendix 7).

### Joint space width narrowing

Only seven studies reported the joint space width narrowing after the execution of different treatments. Table 2 shows the outcomes of network meta-analyses for joint space width narrowing. The joint space width narrowing of glucosamine (MD = 0.18 mm, 95%CI: 0.03 mm to 0.34 mm) and chondroitin (MD = 0.15 mm, 95%CI: 0.02 mm to 0.30 mm) was significantly smaller than that of placebo. There was no significant difference in terms of joint space width narrowing between any other two compared groups. Moderate inconsistency was observed. The assessment of the goodness of fit suggested an adequate fit with the posterior mean residual deviance equal to 18.62 (19 data points). Evidence of significant heterogeneity was found in some comparisons, but no publication bias was observed among included studies. (Appendix 6).

### Tolerability and adverse effects

A total of 38 studies reported the withdrawal of patients due to AE; 21 reported SAEs, and 25 reported the number of patients with AE. The results of AEs (including the number of withdrawal due to AE, the number of patients with AE and SAE) and tolerability analysis are presented in Table 3. There was no significant difference in the comparisons between any two treatment options. Meanwhile, no evidence of inconsistency was observed between direct and indirect evidences. The assessment of the goodness of fit suggested an adequate fit with the posterior mean residual deviance equal to 74.70 (79 data points) for withdrawal due to AE, 43.26 (45 data points) for SAEs, and 53.28 (54 data points) for the number of patients with AE.

Six specific kinds of AE were reported by pairwise meta-analysis. The results are shown in Table 4. Celecoxib was the only treatment option exhibiting a higher incidence of GI AE compared with placebo (OR = 1.17, 95%CI: 1.02 to 1.34). After excluding the studies with poor methodological quality, the results did not change significantly. Significant heterogeneity existed in the comparison between chondroitin and placebo for MU AE (p = 0.12, I^2^ = 59%) and skin AE (p = 0.07, I^2^ = 62%). No publication bias was observed among included studies. (Appendix 6).

## Discussion

This network meta-analysis included 54 studies covering 16,427 knee OA patients. The results indicated significant effects of glucosamine plus chondroitin in pain relief and function improvement compared to the placebo group. In addition, this combination group was the only treatment option exhibiting clinically significant improvement from baseline pain and function. The structure-modifying effect of glucosamine, chondroitin, or the two in combination, remains uncertain due to the weakness of evidence. Furthermore, no significant difference was observed among the five treatment options with respect to adverse effects. However, the additional classical meta-analysis showed that celecoxib exhibited a higher rate of GI adverse effect comparing with placebo.

Glucosamine is a major constituent of extracellular matrix macromolecules such as glycosaminoglycans. The relevant mechanisms of glucosamine include the anti-inflammatory effect, pro-anabolic effect, promotion of osteoblast proliferation, and inhibition of catabolic intermediates^32^,^33^,^34^. Chan and colleagues reported that glucosamine plus chondroitin showed complementary anti-catabolic and anti-inflammatory effects when compared with the use of glucosamine or chondroitin alone^35^,^36^,^37^. This is consistent with our finding, i.e., glucosamine plus chondroitin was the only treatment exhibiting clinically significant improvement from baseline pain and function. Chondroitin, a kind of glycoaminoglycan, is a component of the aggrecan structure which makes up the articular cartilage. The anti-inflammatory, anabolic, anti-catabolic, anti-apoptotic and anti-oxidant effects of chondroitin have been widely reviewed^33^,^34^. However, compared with placebo, the present study neither proved a function improvement effect, nor a clinically significant improvement of chondroitin from baseline function.

Celecoxib has been confirmed by our previous study as an effective treatment in pain relief and function improvement for OA^11^. However, this network meta-analysis further indicated that celecoxib did not show any clinically significant improvement from baseline function. In addition, the existing evidence cannot prove the structure-modifying effect of celecoxib due to the limited number of included trials. Although celecoxib was not significantly different from glucosamine plus chondroitin, glucosamine, chondroitin, or placebo with respect to the three major adverse effects, the additional classical meta-analysis showed that celecoxib exhibited a higher rate of GI adverse effect comparing with placebo. According to the results of our previous study, 200 mg QD exhibited a significantly higher incidence of GI adverse effect in comparison with placebo^11^. Thus, 100 mg BID oral celecoxib might be considered as the preferred dosage for the treatment of knee OA.

To our best knowledge, this is the first network meta-analysis that compares glucosamine, chondroitin, and the two in combination with celecoxib or placebo for the treatment of knee OA. Both direct and indirect comparisons were taken into account while fully preserving randomization. In addition, this study not only examined the treatment effects of glucosamine, chondroitin and the two in combination, but also compared their effects with the effects of celecoxib, the most widely-used selective NSAID at present. This network meta-analysis was designed specifically in accordance with two latest high-quality RCTs^10^,^38^. Nevertheless, limitations of the present study should also be acknowledged. Firstly, because of the limited number of included studies, the some AEs were not combined for network meta-analysis, but combined for the classical meta-analysis. Secondly, a moderate level of inconsistency was observed for pain and joint space width narrowing. This may be caused by some unknown confounding factors which may affect the results of indirect comparisons. The limited number of studies that investigated the combination of glucosamine and chondroitin may be one of the reasons. Thirdly, variations in treatment duration, dosage (especially for glucosamine and chondroitin), brand, final follow-up time point, and sample size may contribute to the significant heterogeneity of evidence. Particularly, the possible duration-response pattern may exert an impact on the performance of these drugs. Fourthly, more high-quality RCTs with longer follow-up time interval are needed to confirm these drugs’ safety, especially the cardiac-vascular and renal safeties.

The network meta-analysis performed by Wandel *et al.* in 2010 suggested that glucosamine, chondroitin, and the two in combination did not mitigate joint pain or imposed an impact on narrowing of joint space^12^. The conclusion of this study played a crucial role in shifting the attitudes of several guidelines with respect to the effect of glucosamine and chondroitin in the treatment of OA. However, some worthwhile issues should be mentioned. The overall difference in pain intensity based on a summary of all time points was −0.4 cm on a 10 cm visual analogue scale for glucosamine, −0.3 cm for chondroitin, and −0.5 cm for the two in combination when compared with placebo. The treatment duration in most of the included RCTs exceeded 24 months, but the selected nine time windows (up to 3, 6, 9, 12, 15, 18, 21 months or more) were all less than 24 months. In other words, the overall difference in pain intensity based on a summary of all time points cannot reflect the curative effect after the treatment duration. The effects of those treatments were probably underestimated. Second, Wandel’s study searched electronic databases from inception to June 2009 and only included the large-scale RCTs with more than 200 patients. Their evidence was limited because of the very small number of included RCTs (only ten), especially for the combination group, which is deemed as the most promising treatment depending on only a single RCT. Such results may not be reliable and lack of external validity. Last, in their study, only pain relief and the structure-modifying effect, but not the function improvement effect, were measured.

High-quality RCTs and observational studies could be complementary to each other. The efficacy evidence of a treatment in clinical trial often lacks of generalizability, while observational studies can report a more realistic expectation for treatment benefits in a real-world environment^39^. However, some scholars argued that the internal validity of trails is more important than generalizability^40^. Yang and colleagues conducted an analysis with the marginal structural model and their results indicated that the use of glucosamine and chondroitin did not relieve symptoms or modify disease progression (JSW) in a 4-year follow-up^41^. Martel-Pelletier and colleagues conducted a first-line analysis, which suggested that the use of glucosamine and chondroitin with or without NSAIDs over 24 months showed a disease-modifying effect^42^. Another analysis conducted by Bertin and colleagues used a French database suggested a significant NSAID-sparing effect of glucosamine in patients with knee OA^43^.

The evidence provided by this study could support the conclusion of the latest RCT that glucosamine and chondroitin have comparable efficacy over celecoxib in pain relief and function improvement^10^. This study also suggested that the combination of glucosamine and chondroitin is the only treatment exhibiting clinically significant improvement from baseline pain and function. In recent years, changes in attitudes about the use of glucosamine and chondroitin in the treatment of OA have been witnessed. For example, the Irish national trend in prescribing of glucosamine increased significantly from 2002 to 2009, but then started decreasing in 2010 and 2011 under the influence of the international guidelines^44^. Before 2010, all available guidelines supported the use of glucosamine and chondroitin in symptomatic relief, and indicated that glucosamine and chondroitin may have a structure-modifying effect^45^,^46^,^47^,^48^. In 2010, the turning point came with the OARSI recommendations which suggested that the effect size for pain relief from glucosamine and chondroitin diminished^49^. In the latest available guidelines, glucosamine and chondroitin have even been labeled as not recommended^2^,^3^,^4^. Based on the research findings of the present study, we do not oppose the use of glucosamine plus chondroitin for the treatment of knee OA in the future clinical practice, especially for patients with moderate-to-severe knee pain^50^. In addition, we strongly recommend that the future guidelines should reconsider the use of glucosamine and chondroitin for the treatment of knee OA.

In view of the limited number of direct evidences that compare glucosamine plus chondroitin with others options, further high-quality RCTs involving direct comparisons, particularly industrial independent trails that examine the structure-modifying effects, are desired. Some additional questions should be addressed as well, e.g., what is the best treatment duration (shortest in the case of effective). Moreover, the follow-up duration of future studies should be extended to determine whether the treatment effects may diminish with time. Apart from these areas, there is a particularly important suggestion with respect to the target population. On the one hand, subsequent RCTs need to test the effect of the combination of glucosamine and chondroitin on knee OA patients with moderate-to-severe pain. On the other hand, future studies should pay more attention to the early-stage OA^1^.

## Conclusion

The present study provided evidence for the symptomatic efficacy of glucosamine plus chondroitin in the treatment of knee OA.

## Additional Information

**How to cite this article**: Zeng, C. *et al.* Effectiveness and safety of Glucosamine, chondroitin, the two in combination, or celecoxib in the treatment of osteoarthritis of the knee. *Sci. Rep.* **5**, 16827; doi: 10.1038/srep16827 (2015).

## Figures and Tables

**Table 1:**
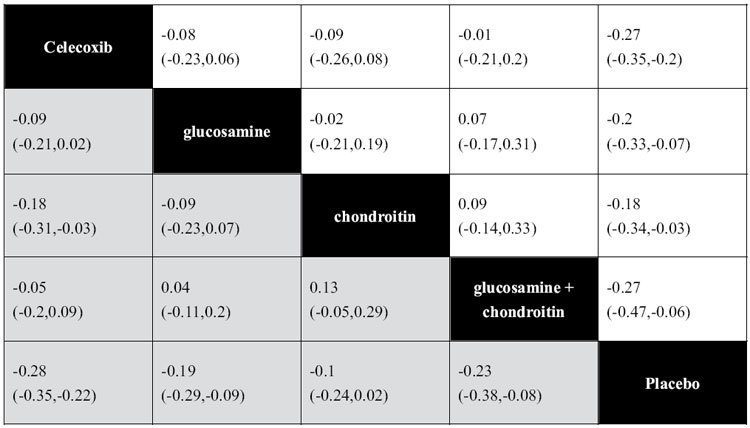
Network meta-analyses comparison between results of pain difference (white) and function difference (grey) at last follow-up time point from the baseline.

Data were pooled standard mean difference (SMD) and its related 95%CI.

**Table 2:** Network meta-analyses comparison between results of JSW.

Comparison	JSW
Celecoxib vs GS	−0.08 (−0.57,0.43)
Celecoxib vs CS	−0.07 (−0.54,0.42)
Celecoxib vs GS+CS	−0.08 (−0.59,0.48)
Celecoxib vs Placebo	0.22 (−0.25,0.69)
GS vs CS	0.01 (−0.30,0.31)
GS vs GS+CS	0.00 (−0.33,0.40)
GS vs placebo	0.30 (0.00,0.55)
CS vs GS+CS	−0.01 (−0.35,0.36)
CS vs placebo	0.29 (0.04,0.50)
GS+CS vs placebo	0.30 (−0.09,0.63)

Data were pooled standard mean difference (SMD) and its related 95%CI.

GS, glucosamine; CS, chondroitin; JSW, joint space width.

**Table 3:** Network meta-analyses comparison between results of adverse events.

Comparison	Withdrawal due to adverse events	Serious adverse events	Number of patients with adverse events
GS vs placebo	0.86 (0.60,1.22)	1.03 (0.21,2.95)	1.12 (0.77,1.61)
CS vs placebo	1.33 (0.91,1.85)	1.46 (0.55,3.15)	1.08 (0.77,1.49)
GS+CS vs placebo	1.01 (0.6,1.59)	1.07 (0.29,2.95)	1.01 (0.62,1.52)
Celecoxib vs Placebo	1.04 (0.86,1.26)	1.16 (0.72,1.83)	0.97 (0.82,1.12)
CS vs GS	1.58 (0.97,2.44)	2.21 (0.34,7.61)	1.00 (0.58,1.61)
GS+CS vs GS	1.20 (0.66,1.99)	1.63 (0.18,6.73)	0.93 (0.49,1.59)
Celecoxib vs GS	1.24 (0.82,1.77)	1.77 (0.36,5.73)	0.89 (0.58,1.29)
GS+CS vs CS	0.78 (0.41,1.29)	0.88 (0.16,2.63)	0.96 (0.51,1.64)
Celecoxib vs CS	0.81 (0.54,1.18)	0.97 (0.33,2.39)	0.92 (0.62,1.30)
Celecoxib vs GS+CS	1.09 (0.66,1.69)	1.49 (0.41,3.91)	1.00 (0.67,1.48)

Data were pooled odds ratio (OR) and its related 95%CI.

GS, glucosamine; CS, chondroitin.

**Table 4:** Odds ratio (95%CI) of specific adverse effects between different treatment groups.

Comparisons	GI AE	CV AE	CNS AE	Infection	MU AE	Skin AE
Celecoxib vs. placebo	1.17 (1.02, 1.34)	1.12 (0.66, 1.90)	0.95 (0.81, 1.11)	1.02 (0.85, 1.21)	0.77 (0.60, 1.00)	0.84 (0.54, 1.30)
GS vs. placebo	0.83 (0.62, 1.11)	0.84 (0.54, 1.30)	0.92 (0.58, 1.44)	1.15 (0.81, 1.62)	1.33 (0.88, 2.00)	0.61 (0.32, 1.14)
CS vs. placebo	0.76 (0.56, 1.03)	1.17 (0.46, 2.98)	0.88 (0.43, 1.78)	1.08 (0.72, 1.62)	0.57 (0.10, 3.43)	1.76 (0.21, 14.73)
Celecoxib vs. GS	1.41 (0.79, 2.53)	—	—	—	—	1.75 (0.41, 7.52)

GS, glucosamine; CS, chondroitin; JSW, joint space width; GI, gastrointestinal; AE, adverse events; CV, cardiovascular; CNS, central nervous system; MU, musculoskeletal.
